# The role of personality traits and leisure activities in predicting wellbeing in young people

**DOI:** 10.1186/s40359-022-00954-x

**Published:** 2022-11-04

**Authors:** Sarah L. Asquith, Xu Wang, Daniel S. Quintana, Anna Abraham

**Affiliations:** 1 School of Humanities and Social Sciences, Leeds Beckett University, City Campus, Leeds, LS1 3HE UK; 2 Norwegian Centre for Mental Disorders Research (NORMENT), Division of Mental Health and Addiction, Oslo University Hospital & Institute of Clinical Medicine, University of Oslo, Oslo, Norway; 3 Department of Psychology, University of Oslo, Oslo, Norway; 4 NevSom, Department of Rare Disorders & Disabilities, Oslo University Hospital, Oslo, Norway; 5 Department of Educational Psychology, Mary Frances Early College of Education, University of Georgia, Athens, USA; 6 Torrance Center of Creativity and Talent Development, Mary Frances Early College of Education, University of Georgia, Athens, USA

**Keywords:** Wellbeing, Personality, Leisure activities, Adolescents, Young adults, Creativity

## Abstract

**Background:**

The relationship between wellbeing and personality has been studied extensively, but few studies have examined these in the period of adolescence and emerging adulthood. Moreover, the influence of contextual factors such as engagement in leisure activities are rarely considered.

**Methods:**

The present study employs a combination of frequentist and Bayesian analyses to evaluate the concurrent impact of personality traits and leisure activities on five conceptions of wellbeing (life satisfaction; positive affect; negative affect; mental health; flourishing) in three cohorts of young people (aged 14–15; 16–17; 18–20 years).

**Results:**

Personality traits were the only significant predictors of life satisfaction and negative affect, but leisure activities in the form of socialising or physical activity, in addition to personality traits, predicted positive affect, mental health and flourishing. Neuroticism was the largest predictor of wellbeing overall, whereas conscientiousness was the most consistent. Lower levels of wellbeing were also associated with higher levels of creative potential.

**Conclusions:**

The study not only confirms the importance of personality traits as predictors of wellbeing in adolescents and young adults, but also indicates the necessity to consider the impact of leisure activities in different conceptions of wellbeing. The negative relationship between creative potential and wellbeing is in line with the literature which shows a link between mental illness, particularly at subclinical levels, and creativity.

**Supplementary Information:**

The online version contains supplementary material available at 10.1186/s40359-022-00954-x.

## Introduction

Wellbeing is a multi-dimensional construct that describes positive mental feeling and functioning, and not merely the absence of mental illness [1]. It has been defined in terms of two approaches which have their roots in Aristotelian philosophy: hedonic or emotional wellbeing, which is linked to the concept of happiness, and eudaimonic or psychological wellbeing, which relates to positive functioning and achievement of human potential [2–4]. Emotional wellbeing reflects people’s evaluation of their lives and has two components: life satisfaction, a cognitive appraisal of all aspects of a person’s life; and affective wellbeing, a balance of positive affect over negative affect [1]. Psychological wellbeing comprises factors such as self-acceptance, positive relations with others, autonomy, environmental mastery, purpose in life, and personal growth [4]. Keyes [5] has proposed that these approaches be combined in a definition of mental health which comprises emotional wellbeing and positive functioning. Positive functioning consists of a private and personal evaluation of psychological wellbeing, and a more public and social evaluation of functioning in society and community (social wellbeing). Wellbeing research based on this definition has found that emotional wellbeing is correlated with positive affect, negative affect and life satisfaction, psychological wellbeing with aspects such as self-esteem, and social wellbeing with social engagement and political participation [6]. More recently, researchers proposed the concept of flourishing, which incorporates both positive feeling and positive functioning, and corresponds to very high levels of subjective wellbeing (e.g., [5, 7]). Whereas in some self-report scales higher flourishing is represented by higher scores (e.g., [7]), others use diagnostic criteria and thresholds (e.g., [5]). Historically, wellbeing research has focused on measures related to emotional wellbeing, rather than a measure which combines positive feeling and functioning [2].

### Predictors of wellbeing: individual factors

Personality traits are often considered the strongest individual predictors of emotional wellbeing, and the Big Five model of personality is the most commonly used [8]. Within this model, relationships have been found between all five traits and measures of wellbeing, although these relationships differ by trait, and conception of wellbeing. Meta-analyses have revealed neuroticism to be the strongest predictor of negative affect and life satisfaction, and extraversion the strongest predictor of positive affect. The other three personality traits, conscientiousness, agreeableness and openness are also related to wellbeing, but the strength of the relationship and the relative importance of each trait are less consistent and vary by measure of wellbeing [8–10]

There has been less research on the relationship between personality traits and psychological wellbeing and flourishing. Overall, neuroticism, extraversion and conscientiousness are stronger predictors than agreeableness and openness [11, 12]. Studies in children and young people have mainly focused on the personality traits of neuroticism and extraversion and on emotional wellbeing and report similar relationships to those in adult populations [13, 14].

### Predictors of wellbeing: leisure activities

The period of adolescence and young adulthood is characterised by transitions between school, higher education and work, and increasing independence and personal responsibility across cultures, and these transitions can have implications for ongoing health and wellbeing [15]. Contextual factors that have been studied in relation to wellbeing in adolescents and young adults include family and peer relationships as well as school environment [16, 17]. Leisure activities such as creative hobbies, physical activity and socialising offer young people opportunities for autonomy and identity exploration and can contribute both positively and negatively to wellbeing [18, 19].

The effects of physical activity on wellbeing have been studied extensively. In children and adolescents, it is associated with lower levels of anxiety, depression and socio-emotional difficulties, as well as higher self-esteem and cognitive functioning, and higher psychological wellbeing in adults 15 years later [20, 21]. Physical activity might improve wellbeing through improvements in physical self-perceptions (e.g., competence, appearance and self-concept) and self-esteem [22] and when carried out in groups or teams may produce social interactions which enhance social support and a sense of belonging [23].

In contrast, sedentary activities, which include TV watching and screen-based media use (e.g., social media, gaming) are negatively associated with wellbeing, happiness and life satisfaction, and positively associated with socio-emotional difficulties in children and adolescents [24, 25]; see [26] on digital technology use specifically). These negative effects may be explained by solitary activities leading to feelings of isolation, cultural messages via the media affecting mental health related behaviours, and excessive screen-based activities displacing other beneficial activities such as physical activity and sleep [25].

Family and peer relationships are important factors in wellbeing among adolescents and young adults [16]. Engaging in activities with others and spending increased time with friends have been associated with higher life satisfaction in such samples [27]. Newman, Tay [28] have proposed that social activities contribute to subjective wellbeing by meeting our needs for affiliation.

### Wellbeing and creativity

Despite the widespread use of artistic participation to influence a range of health outcomes in adults and children, the relationship between wellbeing and leisure engagement in creative activities has received limited empirical attention. One study of university students found that creative activity on one day predicted higher activated positive affect and flourishing the following day [29]. Conversely, another study with adolescents found that engagement in artistic hobbies was associated with lower wellbeing in the form of psychological distress and negative mood [30].

The limited evidence therefore does not indicate clearly whether creativity is positively or negatively associated with wellbeing and one factor to take into consideration is the distinction between creative potential (as typically measured by divergent thinking tasks) and creative practice (as assessed via self-report questionnaires). On one hand, abundant evidence indicates a positive association (albeit a modest one) between creative potential and subclinical levels of mental illness [31] [32]. On the other hand, creative practice, particularly art therapy, is sometimes a component of the treatment for mental illness. Meta-analyses of the relationship have shown positive mood to have a positive effect on creativity [33]. Indeed, engagement with the arts is often associated with improvements in physical and mental health, and enhanced wellbeing [34].

There has been little research into creative potential, engagement in creative practice/hobbies, and their relationship with wellbeing in young people. In adults, creative potential is associated with life satisfaction as well as purpose and meaning in life [35] and subjective wellbeing [36]. Creative personality traits and engagement in everyday creative acts is associated with the personal growth aspect of psychological wellbeing [37, 38].

### The current study

The aim of the current study was to explore the predictors of wellbeing in young people, aged 14–20. It formed part of a larger study which also examined predictors of creativity in young people [39]. The study reported here examines five measures of wellbeing which include both hedonic/emotional and eudaimonic/psychological conceptions: life satisfaction, positive affect, negative affect, mental health, and flourishing. The predictors included both individual factors, specifically five personality traits, and contextual factors in the form of four leisure activities. We also explored whether there were differences in levels of wellbeing and its predictors across the three age groups, as well as the relationship between wellbeing and creativity. A combination of frequentist and Bayesian approaches were employed in the data analyses to gain more reliable and deeper insights by comparing the results of the two approaches.

## Method

### Participants

Participants were recruited in three cohorts. Cohorts 1 and 2, aged 14–15 and 16–17, were drawn from secondary schools and colleges in North and West Yorkshire, UK. We contacted all schools and colleges in five towns and cities in the region, and 11 institutions out of 132 agreed to take part. We offered participating schools an academic workshop for their students in return for their involvement. Cohort 3, aged 18–20, was recruited from first year university students at the lead author’s institution, the majority of whom were Psychology undergraduates. Participants could enter a prize draw for cinema vouchers, and university students could opt for course credit. Sociodemographic information associated is presented in Table 1. Data was collected from 437 participants. Fifteen participants were excluded from cohort 2 as they were older than 18, so that the age groups of cohort 2 and cohort 3 did not overlap. Three were excluded as they generated too small a number of responses to be measured in one of the creative potential tasks. Ten participants were excluded as their scores in the intelligence task were below the range that could be translated to IQ (cohort 1 = 6, cohort 2 = 3, cohort 3 = 1). The final sample consisted of 409 participants (cohort 1 = 134, cohort 2 = 204, cohort 3 = 71). See Tables 2 and 3 for the descriptive statistics of the variables.

**Table 1:** Sociodemographic information of the sample

		Cohort 1 (*n* = 134)	Cohort 2 (*n* = 204)	Cohort 3 (*n* = 71)	All (*N* = 409)
Age	Mean	14.91	16.91	19.00	16.62
	SD	0.32	0.41	0.59	1.47
Gender	Male	29	47	4	80
	Female	104	157	67	328
	Non-binary	1	0	0	1
Ethnicity	English/Welsh/Scottish/Northern Irish/British	74	130	57	261
	Irish	0	0	2	2
	Any other white background	1	15	2	18
	White and Black Caribbean	0	3	0	3
	White and Black African	1	0	0	1
	White and Asian	3	3	2	8
	Any Other Mixed/Multiple Ethnic Background	4	1	0	5
	Indian	5	10	4	19
	Pakistani	28	25	1	54
	Bangladeshi	8	4	0	12
	Chinese	2	1	0	3
	Any Other Asian Background	4	3	2	9
	Caribbean	0	1	0	1
	African	1	6	1	8
	Any Other Black/African/Caribbean background	1	1	0	2
	Any Other	0	1	0	1
	Missing	2	0	0	2

There was a significant difference in the ethnic mix of the cohorts, χ^2^ (30, *N* = 407) = 61.86, *p* = .001. Cohort 3 had a larger proportion of English/Welsh/Scottish/Northern Irish/British participants, and a smaller proportion of Pakistani participants, than both cohorts 1 and 2. Cohort 3 had a higher proportion of participants from an Irish background than cohort 2, and cohort 2 had a higher proportion of participants from any other white background than cohort 1

**Table 2:** Summary of sample sizes, means and SDs of the wellbeing, personality, leisure and creativity variables

Variable	Whole dataset			Cohort 1 (age: 14–15)			Cohort 2 (age: 16–17)			Cohort 3 (age: 18–20)		
	n	Mean	SD	n	Mean	SD	n	Mean	SD	n	Mean	SD
Life satisfaction	408	3.43	0.89	134	3.51	0.93	204	3.37	0.89	70	3.45	0.82
Positive affect	374	21.90	3.76	118	22.47	3.59	188	21.36	4.04	68	22.38	3.02
Negative affect	374	16.45	4.20	118	16.22	4.12	188	16.64	4.24	68	16.35	4.29
Mental health	391	2.74	0.88	127	2.85	0.87	194	2.65	0.89	70	2.78	0.86
Extraversion	408	6.55	2.15	134	6.60	2.03	204	6.47	2.27	70	6.69	2.05
Agreeableness	405	7.18	1.56	131	7.12	1.49	204	7.17	1.66	70	7.30	1.38
Conscientiousness	405	6.11	1.70	133	6.04	1.65	202	6.05	1.77	70	6.43	1.61
Neuroticism	406	7.00	2.00	134	6.68	1.90	202	6.94	2.09	70	7.76	1.75
Openness	409	6.56	2.07	134	6.75	1.91	204	6.44	2.23	71	6.51	1.89
Creative hobbies	281	5.74	6.51	60	7.38	7.97	150	5.87	6.34	71	4.08	5.03
Physical activity	278	5.76	4.85	56	7.53	5.95	151	5.54	4.74	71	4.84	3.70
Socialising	278	10.11	5.85	56	11.52	6.71	151	9.04	5.55	71	11.30	5.34
Sedentary activities	278	23.64	5.89	56	24.35	6.53	151	23.38	5.92	71	22.15	5.14
AUT Fluency	407	4.36	1.67	132	4.09	1.73	204	4.43	1.71	71	4.64	1.36
AUT Overall originality	407	6.42	2.08	132	6.55	2.40	204	6.21	1.88	71	6.79	1.94
AUT Peak originality	407	6.01	3.64	132	5.86	3.71	204	5.89	3.64	71	6.65	3.51
OKC	390	1.23	0.95	129	1.26	0.97	195	1.31	0.92	66	0.97	0.96

**Table 3:** Frequency statistics for mental health category

	Whole dataset *N* = 391		Cohort 1 *n* = 127		Cohort 2 *n* = 194		Cohort 3 *n* = 70	
Flourishing	84	22%	30	24%	35	18%	19	27%
Moderately mentally healthy	264	68%	86	68%	135	70%	43	61%
Languishing	43	11%	11	9%	24	12%	8	11%

### Materials

#### Wellbeing

Participants completed three wellbeing scales. In the Satisfaction with Life Scale Adapted for Children [40], developed to use with children aged 9–14 years, participants respond on a 5-point scale (1 = *disagree a lot*, 5 = *agree a lot*) to five statements (e.g., ‘I am happy with my life’), providing a single life satisfaction score (Cronbach’s α = 0.848). The Scale of Positive and Negative Experience [7] is a 12-item scale that measures positive and negative feelings and has been used with adolescent and adult samples. Participants report how much they have experienced certain feelings (e.g., happy, afraid) over the past four weeks on a 5-point scale (1 = very rarely or never, 5 = very often or always) providing total scores for positive and negative affect (Cronbach’s α = 0.839 and 0.800, respectively). The Mental Health Continuum-Short Form (MHC-SF; [41] is a 14-item scale which measures emotional, social and psychological wellbeing and has been used with adults and adolescents aged 12–18 years. Participants are asked how they have felt over the past month with a series of statements (e.g., ‘interested in life’) and respond on a six-point scale (0 = never, 5 = every day). The data is coded to produce a total mental health score (Cronbach’s α = 0.890) and a categorical variable which summarises three levels of mental health: ‘flourishing’, ‘languishing’ or ‘moderately mentally healthy’ based on the frequency of their experienced wellbeing symptoms.

#### Personality

A ten-item version of the Big Five Inventory was used (BFI-10; [42] in light of the age range of the young participants. The scale has two items for each trait. Participants respond on a 5-point scale (1 = *disagree strongly*, 5 = *agree strongly*), resulting in scores of 2–10 for extraversion, agreeableness, conscientiousness, neuroticism and openness. The Spearman-Brown split half statistic was used to determine the reliability: Extraversion 0.750, Agreeableness 0.237, Conscientiousness 0.496, Neuroticism 0.512 and Openness 0.615. The statistic of 0.237 for Agreeableness is low and this should be borne in mind in interpreting the results of the inferential statistics.

#### Leisure questionnaire

The research team developed a questionnaire (see Additional file 1) asking participants what they did in their spare time from examples of questionnaires within the leisure literature [43–46]. It covered four main areas: creative hobbies and interests, sports and physical activity, socialising, and sedentary/relaxing activities. Participants were asked to indicate how often they had done the activities listed within each area over the last month. Scores for engagement were calculated based on the number of activities ticked or listed by the participants, and how often they engaged in them per week (less than once = 0.5, 1–2 days = 1.5, 3–4 days = 3.5, 5–6 days = 5.5, and every day = 7).

#### Creativity task 1

The Alternate Uses Task (AUT) [47] was employed where participants were given two minutes per item to generate many unusual uses for three objects (newspaper, shoe, and paperclip). The responses for all age groups were scored together and three dependent measures were derived from the responses. Fluency was calculated as the average number of uses generated for the three items. Overall originality was calculated based on the average frequency of the uses generated by the participant within the whole dataset [48, 49]. The data for cohorts 1 and 2 was highly skewed (z-score =  − 10.61 and − 18.44 respectively), and so the data was transformed for all three cohorts, using a reflected inverse approach, overall originality = 1/(1 − average originality index). Peak originality was calculated as the number of responses given by the participant that were generated by only 10% or less of the participants [48].

#### Creativity task 2

The overcoming knowledge constraints task (OKC) requires participants to invent a new toy [50, 51]. The participants were shown three examples of novel toys invented by others before they drew their own invention. The examples contained three common elements: a ball, the use of electronics, and the need for physical exertion. The inventions were scored based on how many of these three elements they contained (0 to 3). For ease of interpretation the scores were reversed so that a higher OKC raw score meant a greater ability to overcome knowledge constraints. As few participants (29/390) achieved a score of 3, the OKC raw score was recoded into a binary variable, OKC (scores of 0 and 1 = 0, scores of 2 and 3 = 1).

### Procedure

The data collection took place in group sessions at schools, colleges or the university. The Local Research Ethics Committee at the lead author’s institution granted permission for the study. Participants recorded their responses in a booklet. The researcher guided the students through the session and the instructions for each task using a standard script and PowerPoint slides. All the questionnaires and stimuli for the sessions were in the booklets, apart from the example drawings for the Overcoming Knowledge Constraints task. The session was 1–1¼ hours in duration. Due to timetabling constraints, participants in cohorts 1 and 2 were not all able to complete all the tasks (Table 2 shows the sample size for each task). Students first provided some information about demographics and their current studies and/or past school results, and then completed the wellbeing measures, the creativity measures, the BFI-10, and the leisure questionnaire. Participants also completed tasks to measure intelligence and executive functions which are not discussed in this paper. Thirty-one students from cohort 2 completed the leisure questionnaire online. To ensure adequate language proficiency to complete the tasks accurately, participants who had not been studying at an English-speaking school for five years or more were excluded from the sample.

### Approach to inferential statistics

Data analysis was carried out using IBM SPSS Statistics version 24 and the JASP software package version 0.8.6.0 [52]. A traditional frequentist framework approach cannot use *p*-values to determine the relative strength of the evidence for a null or alternate hypothesis, or whether a non-significant *p* value represents a null effect or insensitive data, no matter the size of the *p*-value [53–55]. Bayesian hypothesis testing offers a useful alternative, particularly when it comes to interpreting relative support for a null model against an alternative model [54, 55]. Consequently, a Bayesian hypothesis testing approach, unlike a traditional frequentist approach, can facilitate hypothesis falsification. Bayesian statistical inference is certainly not new; however, it has only more recently been adopted in the biobehavioral sciences. Thus, we present both inference approaches for those not familiar with Bayesian hypothesis testing.

We calculated correlations, and performed linear and nominal regression, and ANOVA in the analysis. In the correlation and ANOVA analyses, we have presented the frequentist *p* value and the Bayes factor from the Bayesian analysis. We have discussed first the findings in which we have higher confidence, where the results from both approaches correspond, i.e., both results supported the alternate hypothesis (a *p* value < 0.05 and Bayes factor ≥ 3), or both results supported the null hypothesis (a *p* value > 0.05 and a Bayes factor < 1/3).^1^ We selected a Bayes factor threshold of 3 (and its inverse), as this closely corresponds to a *p* value of 0.05 [58]. A Bayes factor of 3 suggests that an alternative model is three times more favoured than a null model, given the data. Then we have discussed where the results where the two approaches differ. We have interpreted the results as providing partial support when the frequentist statistics give a significant result and the Bayes factor provides only anecdotal support for the alternate hypothesis, or when the frequentist result is not significant, but the Bayes factor provides at least moderate support. We have interpreted the results as mixed when the frequentist statistics give a significant result, but the Bayes factor provides anecdotal support for the null hypothesis, or when the frequentist statistics give a non-significant result, but the Bayes factor provides anecdotal or moderate support for the alternate hypothesis. The Bayesian correlations used the JASP default stretched beta prior width of 0.5. The Bayesian ANOVA used the JASP default r scale fixed effects prior width of 0.5 for the prior distribution.

To analyse the predictors of wellbeing we used linear and nominal regression, and for the linear regression, we have compared the results of the frequentist regression models with the Bayesian regression analysis in JASP, which presents the results of all possible combinations of the covariates. For the Bayesian regression models, we used a Jeffreys-Zellner-Siow prior with a r scale of 0.354. At the time of carrying out the analysis, a Bayesian alternative to frequentist nominal regression was not available in JASP.

## Results

The aim of the study was to explore the predictors of wellbeing in young people aged 14–20. First, we present the results of the regression analysis that show which individual factors and leisure activities predict wellbeing. Second, we explore whether wellbeing or its predictors varied across the three age groups included in the study. Lastly, we examine the relationship between wellbeing and creativity.

### Predictors of wellbeing across all three cohorts

Descriptive statistics are reported in Tables 2 and 3. Correlations between wellbeing and the predictors (individual factors and leisure activities) are shown in Table 4. In order to identify significant predictors for the measures of wellbeing, linear regressions were run, and *p*-values were adjusted with a Bonferroni correction to correct for multiple tests (see Table 5 for a summary). Eight significant predictors were identified: the personality traits extraversion, agreeableness, conscientiousness, neuroticism and openness to experience (individual factors) and engagement in sports/physical activity, socialising, and sedentary activities (leisure activities). Multiple regressions were run to examine the effect of these predictors on wellbeing (see Tables 6 and 7 for the frequentist multiple regressions summaries and Table 8 for the Bayesian multiple regression summary).

**Table 4:** Correlations between wellbeing, personality trait and leisure activities variables

			1	2		3		4		5		6		7		8		9		10		11		12		13	
1	Life satisfaction	*p*	1	**0.615**	**	** − 0.506**	**	**0.633**	**	**0.168**	**	**0.247**	**	**0.205**	**	** − 0.300**	**	**− **0.133	*	**− **0.075		0.096		0.156	*	** − **0.087	
		BF_10_			> 100		> 100		> 100		> 100		> 100		> 100		> 100		2.31		0.16		0.26		2.20		0.21
2	Positive affect	*p*	373	1		** − 0.473**	**	**0.694**	**	**0.265**	**	**0.192**	**	**0.151**	*	** − 0.326**	**	** − **0.013		0.000		0.162	*	**0.247**	**	0.003	
		BF_10_					> 100		> 100		> 100		65.13		4.35		> 100		0.07		0.08		2.30		> 100		0.08
3	Negative affect	*p*	373	374		1		** − 0.490**	**	** − 0.181**	**	** − 0.259**	**	** − 0.220**	**	** − 0.366**	**	**0.203**	**	0.125	*	**− **0.149	*	**− **0.158	*	0.068	
		BF_10_							> 100		30.35		> 100		> 100		> 100		> 100		0.58		1.23		1.93		0.14
4	Mental health	*p*	390	358		358		1		**0.350**	**	**0.260**	**	**0.225**	**	** − 0.381**	**	**− **0.047		0.012		0.160	*	**0.304**	**	0.001	
		BF_10_									> 100		> 100		> 100		> 100		0.10		0.08		2.35		> 100		0.08
5	Extraversion	*p*	407	373		373		390		1		0.099	*	0.037		** − 0.381**	**	0.006		**− **0.044		0.131	*	**0.296**	**	0.004	
		BF_10_											0.45		0.08		> 100		0.06		0.10		0.79		> 100		0.08
6	Agreeableness	*p*	404	370		370		389		405		1		0.**179**	**	**− **0.115	*	**− **0.041		0.067		0.066		0.060		**− **0.064	
		BF_10_													41.63		0.88		0.09		0.14		0.14		0.12		0.13
7	Conscientiousness	*p*	404	370		370		388		404		402		1		**− **0.002		**− **0.079		**− **0.061		0.132	*	0.040		**− **0.116	
		BF_10_															0.62		0.02		0.13		0.82		0.09		0.48
8	Neuroticism	*p*	405	371		371		388		405		402		405		1		0.006		0.025		** − 0.174**	*	** − 0.167**	*	0.028	
		BF_10_																	0.06		0.08		4.79		3.50		0.08
9	Openness	*p*	408	374		374		391		408		406		405		406		1		**0.394**	**	0.083		**− **0.093		**0.192**	*
		BF_10_																			> 100		0.20		0.25		12.93
10	Creative hobbies	*p*	280	260		260		273		280		278		278		278		281		1		**0.183**	*	0.053		**0.306**	**
		BF_10_																					7.86		0.11		> 100
11	Physical activity	*p*	277	257		257		270		277		275		275		275		278		277		1		0.156	*	0.136	0.*
		BF_10_																							2.20		0.95
12	Socialising	*p*	277	257		257		270		277		275		275		275		278		276		277		1		0.149	*
		BF_10_																									1.62
13	Sedentary activities		277	257		257		270		277		275		275		275		278		276		277		278		1	

Correlation *r* values are shown above the diagonal, with the *p* values from the frequentist correlations indicated by * *p* < .05 and ** *p* < .001, and the Bayes Factors from the Bayesian correlations. Correlations that have a *p* value < .05, and a BF10 > 3 are indicated in bold. *N* is shown below the diagonal. Precise *p* values are available in a copy of the table in Additional file 2

**Table 5:** Results of simple linear regression to identify predictors of wellbeing variables, *p* values

Variable	Life satisfaction	Positive affect	Negative affect	Mental health	Flourishing/languishing
Extraversion	0.001^a^	< 0.001^a^	< 0.001^a^	< 0.001^a^	< 0.001^a^
Agreeableness	< 0.001^a^	< 0.001^a^	< 0.001^a^	< 0.001^a^	< 0.001^a^
Conscientiousness	< 0.001^a^	0.004^a^	< 0.001^a^	< 0.001^a^	< 0.001^a^
Neuroticism	< 0.001^a^	< 0.001^a^	< 0.001^a^	< 0.001^a^	< 0.001^a^
Openness	0.007	0.808	< 0.001^a^	0.354	0.711
Creative hobbies	0.209	0.996	0.044	0.838	0.041
Physical activity	0.112	0.009	0.017	0.009	0.003^a^
Socialising	0.009	< 0.001^a^	0.011	< 0.001^a^	< 0.001^a^
Sedentary activities	0.150	0.964	0.275	0.990	0.004^a^

^a^indicates predictors which are significant after correcting for multiple tests

**Table 6:** Summary of the frequentist multiple regressions predicting wellbeing from personality and leisure activities

Predictor	Life satisfaction			Positive affect			Negative affect			Mental health		
	B	95% CI	β	B	95% CI	β	B	95% CI	Β	B	95% CI	β
Extraversion	0.02	[**− **0.03, 0.07]	0.04	0.15	[**− **0.08, 0.37]	0.08	0.01	[**− **0.24, 0.25]	0.00	0.06**	[0.02, 0.11]	0.16
Agreeableness	0.10**	[0.04, 0.16]	0.18	0.23	[**− **0.05, 0.50]	0.09	** − **0.57***	[**− **0.87, − 0.27]	**− **0.21	0.08*	[0.02, 0.13]	0.14
Conscientiousness	0.08**	[0.03, 0.14]	0.16	0.36**	[0.10, 0.62]	0.16	** − **0.42**	[**− **0.71, − 0.14]	**− **0.16	0.12***	[0.06, 0.17]	0.22
Neuroticism	** − **0.12***	[**− **0.17, − 0.07]	**− **0.27	** − **0.53***	[**− **0.76, − 0.30]	**− **0.29	0.76***	[0.51, 1.01]	0.36	** − **0.12***	[**− **0.17, − 0.07]	**− **0.28
Openness	** − **0.08***	[− 0.13, − 0.04]	**− **0.20	− 0.19^†^	[− 0.40, 0.02]	**− **0.10	0.50***	[0.27, 0.73]	0.24	− 0.03	[− 0.08, 0.01]	**− **0.07
Physical activity	0.01	[− 0.01, 0.03]	0.03	0.04	[− 0.05, 0.13]	0.06	− 0.06	[− 0.16, 0.04]	**− **0.07	0.01	[− 0.01, 0.03]	0.03
Socialising	0.01	[− 0.01, 0.03]	0.08	0.08*	[0.01, 0.16]	0.13	− 0.03	[− 0.12, 0.05]	**− **0.04	0.03**	[0.01, 0.05]	0.19
Sedentary activities	− 0.01	[− 0.02, 0.01]	**− **0.03	0.02	[− 0.06, 0.09]	0.03	− 0.02	[− 0.10, 0.07]	**− **0.02	0.00	[− 0.01, 0.02]	0.02
*R*^2^	0.24			0.23			0.31			0.32		
*F*	10.15			8.89			13.42			15.20		
*p*	< .001			< .001			< .001			< .001		
N	269			249			249			263		

^†^*p* < .10; *p < .05; **p < .01; ***p < .001

**Table 7:** Summary of the frequentist nominal regressions predicting flourishing and languishing from personality and leisure activities

Predictor	Flourishing				Languishing			
	b (SE)	Odds Ratio	95% CI for Odds Ratio		b (SE)	Odds Ratio	95% CI for Odds Ratio	
			Lower	Upper			Lower	Upper
Extraversion	0.13 (0.10)	1.14	0.94	1.38	− 0.20 (0.10)^†^	0.82	0.67	1.00
Agreeableness	0.14 (0.12)	1.15	0.91	1.45	− 0.07 (0.12)	0.93	0.73	1.18
Conscientiousness	0.36 (0.11)**	1.43	1.16	1.78	− 0.25 (0.14)^†^	0.78	0.60	1.01
Neuroticism	− 0.12 (0.10)	0.89	0.74	1.06	0.20 (0.12)^†^	1.22	0.97	1.53
Openness	− 0.03 (0.09)	0.97	0.82	1.14	0.03 (0.10)	1.03	0.85	1.25
Physical activity	0.08 (0.04)*	1.08	1.01	1.15	0.05 (0.05)	1.05	0.97	1.15
Socialising	0.04 (0.03)	1.04	0.98	1.11	− 0.06 (0.05)	0.94	0.86	1.03
Sedentary activities	0.06 (0.03)*	1.07	1.00	1.14	0.06 (0.04)^†^	1.03	0.99	1.14

The multinomial logistic regression was run with “moderately mentally healthy” as the reference category

*R*^2^ = .25 (Cox & Snell), .30 (Nagelkerke). Model χ^2^(16) = 75.51, *p* < .001, N = 263

^†^*p* < .10; *p < .05; **p < .01

**Table 8:** Summary of the Bayesian regression analysis predicting wellbeing from personality and leisure activities: top five models

Variable	Predictors	BF_10_	*R*^2^
Life satisfaction	1. Agreeableness + conscientiousness + neuroticism + openness	1.59e + 11	0.23
	2. Agreeableness + conscientiousness + neuroticism + openness + socialising	9.25e + 10	0.24
	3. Extraversion + agreeableness + conscientiousness + neuroticism + openness	5.21e + 10	0.23
	4. Agreeableness + conscientiousness + neuroticism + openness + physical activity	4.16e + 10	0.23
	5. Agreeableness + conscientiousness + neuroticism + openness + sedentary activities	3.25e + 10	0.23
Positive affect	1. Conscientiousness + neuroticism + socialising	9.85e + 08	0.20
	2. Agreeableness + conscientiousness + neuroticism + socialising	9.15e + 08	0.21
	3. Conscientiousness + neuroticism + openness + socialising	8.78e + 08	0.21
	4. Agreeableness + conscientiousness + neuroticism + openness + socialising	7.45e + 08	0.22
	5. Extraversion + conscientiousness + neuroticism + socialising	5.64e + 08	0.21
Negative affect	1. Agreeableness + conscientiousness + neuroticism + openness	1.41e + 15	0.30
	2. Agreeableness + conscientiousness + neuroticism + openness + physical activity	7.20e + 14	0.31
	3. Agreeableness + conscientiousness + neuroticism + openness + socialising	4.37e + 14	0.30
	4. Agreeableness + conscientiousness + neuroticism + openness + sedentary activities	3.30e + 14	0.30
	5. Extraversion + agreeableness + conscientiousness + neuroticism + openness	2.68e + 14	0.30
Mental health	1. Extraversion + agreeableness + conscientiousness + neuroticism + socialising	6.56e + 16	0.32
	2. Extraversion + agreeableness + conscientiousness + neuroticism + openness + socialising	2.68e + 16	0.32
	3. Extraversion + agreeableness + conscientiousness + neuroticism + physical activity + socialising	1.37e + 16	0.32
	4. Extraversion + conscientiousness + neuroticism + socialising	1.32e + 16	0.30
	5. Extraversion + agreeableness + conscientiousness + neuroticism + socialising + sedentary activities	1.26e + 16	0.32

BF_10_ shows the Bayes factor in comparison to the null model

Looking first at personality traits, higher neuroticism predicted lower wellbeing for the four continuous measures of wellbeing: neuroticism was a negative predictor of life satisfaction, positive affect, and mental health, and a positive predictor of negative affect (all *p* < 0.001). Higher conscientiousness predicted higher wellbeing for the four continuous measures and the categorical measure of wellbeing: conscientiousness predicted life satisfaction, positive affect and mental health positively, and negative affect negatively (all *p* < 0.01), and additionally predicted flourishing (*p* = 0.001). Higher agreeableness also predicted higher wellbeing for three of the measures: it predicted life satisfaction and mental health positively, and negative affect negatively (all *p* < 0.01). Higher openness predicted lower wellbeing in two measures: it predicted life satisfaction negatively and negative affect positively (all *p* < 0.001). Extraversion only positively predicted mental health (*p* = 0.01).

Turning next to leisure activities as predictors of wellbeing, higher levels of physical activity predicted higher wellbeing for only one of the measures, flourishing (*p* = 0.031). Higher levels of socialising predicted higher wellbeing for two of the outcomes: positive affect (*p* = 0.025) and mental health (*p* = 0.002). Engagement in sedentary activities predicted flourishing (*p* = 0.038) but as the confidence interval included an odds ratio of one, this result needs to be interpreted with caution.

Bayesian regression analysis allows us to examine the effect of these predictors further by allowing identification of which of the possible combinations of the predictors produces the best model. With eight predictors in these models, the results tables are very large, so only the top five models are presented in Table 8. The Bayes factors (BF_10_ in Table 8) represent the comparison to the null model and the results show that multiple models were noteworthy predictors of wellbeing. In all cases, the best model contains the same predictors that were significant in the frequentist multiple regressions.

### Differences between the Age Cohorts in Wellbeing and its Predictors

Looking first at wellbeing, both the frequentist and Bayesian analyses indicate no noteworthy differences between the cohorts in most of the measures of wellbeing: life satisfaction, negative affect and mental health, (all *p* > 0.05, BF_10_ < 0.33, see Table 9), and no difference between the cohorts in the proportion of participants who were flourishing or languishing (χ^2^(4) = 3.87*, p* = 0.426). There was however partial support for a difference in positive affect between the cohorts: it was supported by the frequentist analysis (*p* = 0.021), and there was a significant quadratic trend (*F*(1, 371) = 7.30, *p* = 0.007, *η*^2^ = 0.02) which indicated that levels of positive emotions were lower in the 16–17 age group than the 14–15 and 18–20 groups. However, this was supported only anecdotally by the Bayesian analysis (BF_10_ = 1.26).

**Table 9:** Differences between the three cohorts for wellbeing and predictor variables using null hypothesis significant testing (NHST) and Bayesian analyses

Variable	NHST	Bayes factor	
Life satisfaction	Non-significant (*F*(2, 405) = 0.94, *p* = .393)	0.07	Strong support for the null hypothesis
Positive affect	Significant (*F*(2, 371) = 3.88, *p* = .021)	1.22	Anecdotal support for the alternate hypothesis
Negative affect	Non-significant (*F*(2, 371) = 0.38, *p* = .683)	0.05	Strong support for the null hypothesis
Mental health	Non-significant (*F*(2, 388) = 2.10, *p* = .124)	0.23	Moderate support for the null hypothesis
Flourishing/languishing	Non-significant (χ^2^(4) = 3.87*, p* = .426)		
Extraversion	Non-significant (*F*(2, 405) = 0.33, *p* = .722)	0.04	Strong support for the null hypothesis
Agreeableness	Non-significant (*F*(2, 402) = 0.30, *p* = .739	0.04	Strong support for the null hypothesis
Conscientiousness	Non-significant (*F*(2, 402) = 1.45, *p* = .235)	0.11	Moderate support for the null hypothesis
Neuroticism	Significant (*F*(2, 194.69) = 8.50, *p* < .001)*	16.73	Strong support for the alternate hypothesis
Openness	Non-significant (*F*(2, 406) = 1.00, *p* = .370)	0.07	Moderate support for the null hypothesis
Creative hobbies	Significant (*F*(2) = 4.35, *p* = .014)	1.91	Anecdotal support for the alternate hypothesis
Physical Activity	Significant (*F*(2, 125.45) = 4.37,* p* = .015)*	4.39	Moderate support for the alternate hypothesis
Socialising	Significant (*F*(2, 275) = 5.84,* p* = .003)	8.55	Moderate support for the alternate hypothesis
Sedentary activities	Non-significant (*F*(2, 275) = 2.26, *p* = .106)	0.31	Moderate support for the null hypothesis

* Welch’s *F*

Looking next at personality across the three cohorts, the results suggested that there were no differences in four of the five traits. There was however a difference in neuroticism, which was supported by both the frequentist and Bayesian analysis (*p* < 0.001, BF_10_ > 10). Analysis indicated a significant linear trend, (*F*(1, 405) = 12.16, *p* = 0.001, *η*^2^ = 0.03), with neuroticism increasing with age. However, there were fewer male participants in cohort 3 than in the other two. A chi-square test found a significant difference in gender splits between the cohorts, χ^2^ (4, *N* = 409) = 12.81, *p* = 0.012, with 6% of participants in cohort 3 being male, compared to 22% and 23% in cohorts 1 and 2 respectively, which suggests that the difference is explained by a difference in gender distribution across cohorts.

**Fig. 1:**
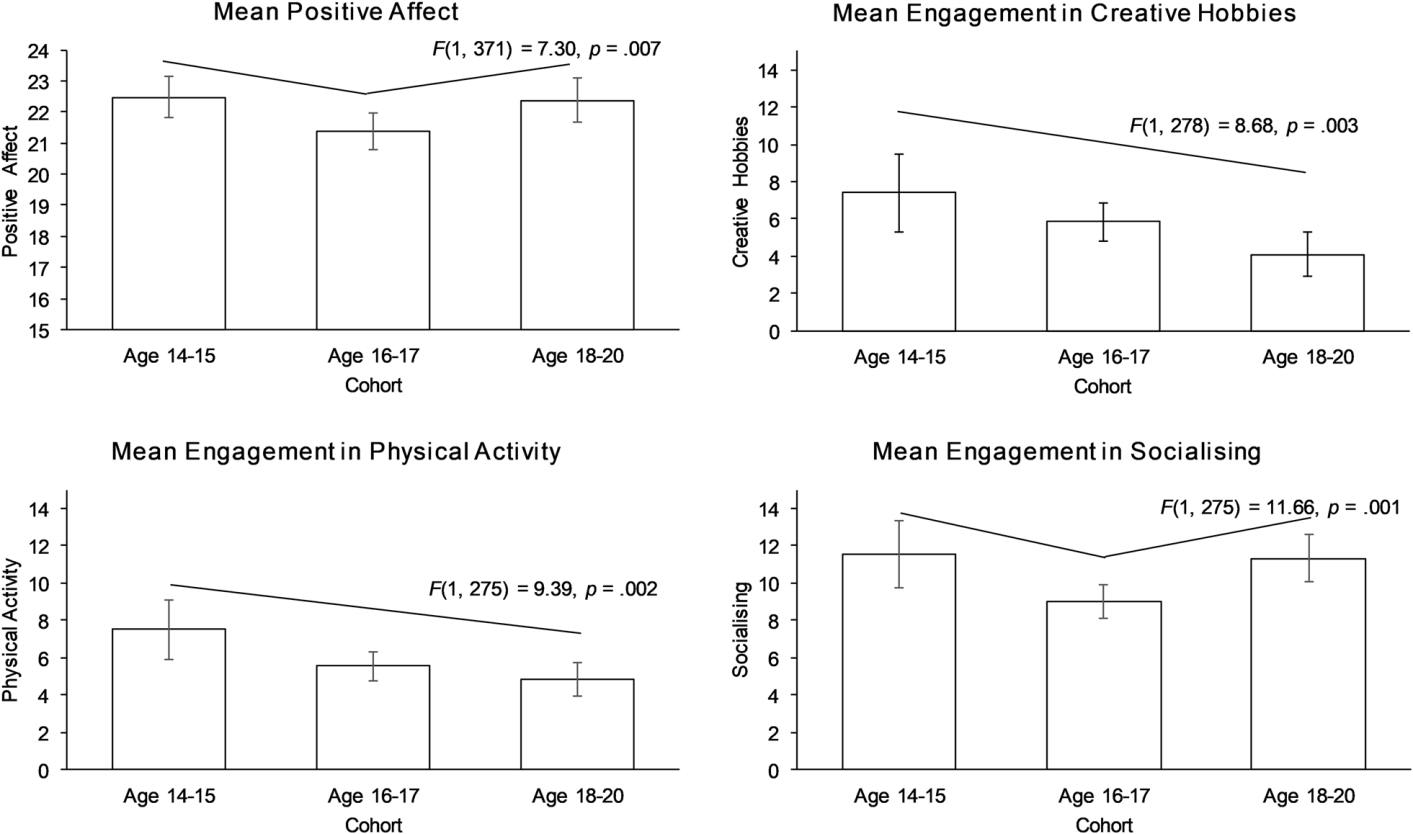
Means of positive affect and engagement in leisure activities by cohort

Moving on to consider leisure activities across the three cohorts, the results suggested some different patterns of engagement, with declines in creative hobbies and physical activity, and a U-shaped pattern in socialising (see Fig. 1). Engagement in creative hobbies appeared to decline with age: a difference was supported by the frequentist analysis (*p* = 0.008) and there was a significant linear trend (*F*(1, 278) = 8.68, *p* = 0.003, η^2^ = 0.03), but the difference was supported only anecdotally by the Bayesian analysis (BF_10_ = 1.91). Similarly, physical activity appeared to decline with age; a difference across the cohorts was supported by both the frequentist and the Bayesian analysis (*p* = 0.015, BF_10_ = 4.39), and analysis revealed a significant linear trend (*F*(1, 278) = 9.39, *p* = 0.002, η^2^ = 0.03). There was a difference in engagement in socialising between the cohorts, and this was supported by both the frequentist and Bayesian analysis, (*p* = 0.003, BF_10_ = 4.39; see Fig. 1); there was a significant quadratic trend (*F*(1, 277) = 11.66, *p* = 0.001, η^2^ = 0.04), indicating that the middle age group, aged 16–17, spent less time socialising than the younger and older age groups. This suggests that while engagement in creative hobbies and physical activity may decline overall from age 14/15 to age 18/20, engagement in socialising dips at age 16/17 before rising again by age 18/20.

### The Relationship between Creativity and Wellbeing in Young People

Correlation analysis revealed a negative relationship between wellbeing and creative potential. Higher levels of negative affect were associated with higher performance on two measures of creative potential: fluency and peak originality, and although the size of the correlations is modest (*r* = 0.16 and 0.15 respectively; see Table 10) this was supported by both the frequentist and Bayesian approaches (*p* < 0.01, BF_10_ > 3). There was also mixed evidence for an association between lower levels of mental health and higher levels of fluency. This was supported by the frequentist analysis (*r* = −0.11, *p* = 0.027), but not the Bayesian analysis (BF_10_ = 0.73).

**Table 10:**
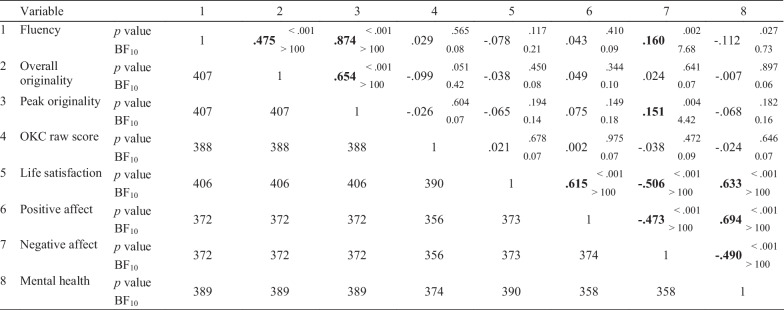
Correlations between creativity and wellbeing variables

*Notes:* Correlation *r* values are shown above the diagonal, with the *p* values from the frequentist correlations, and the Bayes Factors from the Bayesian correlations. Correlations that have a *p* value < .05, and a BF_10_ > 3 are indicated in bold. N is shown below the diagonal

## Discussion

We found that all the Big Five personality traits predicted wellbeing, but the relative importance of the traits differed according to the different measures of wellbeing, and these findings were supported by both the frequentist and Bayesian analyses. Neuroticism was the largest predictor of four out of five measures of wellbeing, life satisfaction, positive and negative affect, and mental health. This is largely consistent with the meta-analyses of DeNeve and Cooper [9] and Steel, Schmidt [8], which found that neuroticism had the largest relationship with life satisfaction and negative affect. McCrae and Costa [10] attributed such effects of neuroticism to temperament as people high in neuroticism are more prone to experiencing negative emotions. The results of the present study confirm the relevance of neuroticism in predicting wellbeing in adolescence and young adulthood.

Conscientiousness was the most consistent predictor of wellbeing, predicting all five conceptions of wellbeing and it was the only personality trait that predicted flourishing, the measure of very high levels of subjective wellbeing. This finding is consistent with previous research showing associations between conscientiousness and life satisfaction, positive affect and negative affect [8], and extends the importance of this trait in young people to mental health and flourishing. McCrae and Costa [10] suggested that conscientiousness might contribute to wellbeing through instrumental effects: that individuals high in conscientiousness are efficient and hard-working, and achievement at work contributes to positive affect and life satisfaction. In the present sample, these instrumental effects may operate similarly through academic achievement. Conscientiousness may also be adaptive in meeting norms and standards that are set by others (such as parents and teachers) which marks experiences for young people in school and university [59]. In this study it is notable that conscientiousness contributed to (a) life satisfaction, positive affect and negative affect (emotional wellbeing), (b) social and psychological wellbeing (represented by the mental health construct), and (c) flourishing. This confirms the importance of a trait that has previously been considered less significant for wellbeing than neuroticism and extraversion [60].

Agreeableness predicted life satisfaction and negative affect but did not predict positive affect, which is consistent with previous research [8]. It also predicted mental health, which includes social and psychological wellbeing. McCrae and Costa [10] suggested that agreeableness operates in a similar way to conscientiousness to contribute to wellbeing: individuals high in agreeableness have positive relationships with family, friends and work colleagues, and these social bonds contribute to positive affect and life satisfaction. Adolescence and young adulthood are periods in which skills are acquired in starting and maintaining relationships but also one in which conflicts may occur. Agreeableness is associated with the selection of appropriate strategies for conflict resolution [61] and may therefore contribute to wellbeing in this way. The gender mix of the sample, being predominantly female, may also have contributed to this finding, as agreeableness has been found to predict life satisfaction in 13 to 19-year-old girls but not boys; for girls, life satisfaction may be affected by consistency between their gender and expression of traits stereotypically associated with femininity [14]. Future research should examine whether the relationship between agreeableness and social and psychological wellbeing differs for girls and boys.

The results relating to extraversion and openness conflict with the literature. Extraversion has been positively associated with life satisfaction, positive affect and psychological wellbeing, and negatively associated with negative affect [8, 9] but in the present study it only predicted mental health. The use of a short measure of personality may have influenced this finding in that the two items for extraversion (“…is reserved (quiet, shy)” and “…is outgoing, sociable”) tap into the sociability dimension of the trait but do not capture positive emotionality or assertiveness. Using a longer measure may therefore have produced similar findings to previous research in finding an association between extraversion and other aspects of wellbeing. Literature on the impact of openness on wellbeing is mixed. McCrae and Costa [10] suggested that people high in openness experience both positive and negative affect more intensely but that openness did not directly affect life satisfaction. Subsequent meta-analyses found that openness is positively related to positive affect but relatively unrelated to negative affect and life satisfaction [8, 60]. In the present study, we found that openness predicted negative affect but not positive affect, and it predicted life satisfaction negatively. Keyes, Shmotkin [62] suggested that openness may increase opportunities for self-fulfilment but also invoke negative feelings and evaluations of one’s life. The balance of positive and negative aspects of this trait for wellbeing warrants further research in young samples.

Broadly speaking, we found larger and more consistent effects for neuroticism, conscientiousness and agreeableness than for extraversion and openness. DeYoung [63, 64] has suggested that the Big Five personality traits can be composed into two higher order factors, stability, comprised of neuroticism, agreeableness and conscientiousness, and plasticity, comprised of extraversion and openness. Strickhouser, Zell [65] found that the three stability traits had similar and larger effects on mental health than the two plasticity traits. Our results seem to follow a similar pattern.

With regard to the influence of the leisure activities that young people engage in on wellbeing, socialising predicted positive affect and mental health. This is consistent with previous research which has found a positive relationship between social activities and life satisfaction in young people [30] and that very happy people had good social relationships [66]. The social activities in the questionnaire included activities such as going to the cinema, and spending time with friends and family, so it is understandable how these could contribute to positive affect and to warm relationships with others, which is included in the mental health construct. Although physical activity has previously been associated with wellbeing in young people [20, 23, 25], in this study, physical activity was only associated with one of the measures of wellbeing, flourishing, which suggests that it differentiated between very high levels of wellbeing and being “moderately mentally healthy”.

Engaging in sedentary activities was also a positive predictor of flourishing. This contrasts with some previous research which has found sedentary activities to be negatively associated with wellbeing in young people [24, 25]. However, the effects of sedentary leisure may be different depending on the type of activity. There is some evidence to suggest a small positive relationship between reading and wellbeing in children and young people [67], listening to music may contribute to wellbeing in young people through emotion regulation and social connectedness [68], and internet use may offer social support [69]. These factors may account for the positive relationship between sedentary activities and flourishing found in this study.

Limited literature has examined how wellbeing varies across the period of adolescence and young adulthood and the findings are mixed. Some studies have found that life satisfaction does not vary with age over this period [70]. Others have shown that high school students had lower life satisfaction than primary school students [71] and that late adolescents (15–18 years) experience lower mental health than early adolescents (12–14 years) [72]. In the current study, there were no differences between the age groups in four of the measures of wellbeing, but the middle age group (16–17) reported lower positive affect than the younger (14–15) and older (18–20) groups. However, it is worth noting that this difference was modest as it was supported only anecdotally by the Bayesian analysis, and the effect size was small. Affect in adolescence may be related to social transitions such as a change of school or college [73], but we did not find lower positive affect in the group who were in their first year at university. Further research in this regard is also warranted.

The decline in physical activity with age reported in the present study is consistent with other studies which found declines in this age group in the US and the UK [74, 75]. In fact, a large body of research supports a relationship between physical activity and mental health [23, 25]. Declines in physical activity over the period of adolescence and young adulthood may therefore be a concern for future levels of wellbeing.

There has been less research into profiles of engagement in creative hobbies across this age span. Auhuber, Vogel [76] found no difference in engagement in activities such as choir/orchestra and theatre/dancing between younger (10–13-year olds) and older age groups (14–18-year olds), but in the broader set of creative hobbies included in this study, we found a decline in engagement with age. It is possible that this decline reflects less time and resources to support these sorts of activities as a result of increasing academic pressure, part time work, and/or living away from home. However as the decline was supported only anecdotally by the Bayesian analysis and the effect size was small, this should be examined further, by creative domain, for instance. In the current study, we also found a dip in engagement in socialising activities in the middle age group (16–17 years). As engagement in socialising activities predicted wellbeing for two of the measures, positive emotions and mental health, the lower engagement in this age group might be relevant for their levels of wellbeing.

There was also evidence for a negative relationship between wellbeing and creative potential, suggesting that ideational fluency (the propensity to generate many ideas) and peak originality (the propensity to generate highly original ideas) was associated with a higher degree of negative affect. This fits Tamannaeifar and Motaghedifard [36]’s finding of a negative relationship between creativity and emotional wellbeing, but contrasts with research that has found positive associations between creativity and some other measures of wellbeing [35–38]. Furthermore, it is in line with the literature showing a link between mental illness, particularly at subclinical levels, and creativity [31, 32].

There are some limitations to the study. As has already been noted in the discussion of extraversion as a predictor of wellbeing, the use of a short form personality measure such as the BFI-10 means that the scale may not reflect the breadth of each trait, nor capture as much of the variance as the full scale [42]. It would therefore be preferable to use a fuller measure when assessment time permits. In addition, as differences have been found in levels of wellbeing and personality traits as a function of gender (e.g., [77, 78]) and age (e.g., [77, 79]), the findings could be affected by factoring in these variables in the statistical analyses. However, as the present study featured a predominantly female sample, which was also disproportionate across the cohorts (see Table 1), gender was not included as an added variable in the analyses on methodological grounds. Age was also not included as a covariate as we collected data from three cohorts, each separated by one academic year. So, age as recorded in this study, is not a continuous variable. Nonetheless, as gender and age are variables of interest in this research area, exploratory analyses including gender and age (using cohort as a proxy for age), the results of which are and these are reported in Additional file 3. The study is also cross-sectional in design, with an unequal sample size in each age group. The findings across the age groups in the study may therefore have been affected by cohort effects. A longitudinal research design would permit a clearer understanding of wellbeing profiles in adolescence and young adulthood.

## Conclusion

Using a novel methodological combination of frequentist and Bayesian approaches, which provided complementary forms of inference, the present study was carried out to examine individual predictors (in the form of five personality traits) and contextual predictors (in the form of four leisure activities) of wellbeing in three cohorts of young people in adolescence and emerging adulthood (aged 14–15, 16–17 and 18–20 years). The personality variables of Conscientiousness, Agreeableness and Extraversion positively predicted wellbeing, while Neuroticism and Openness to Experience negatively predicted wellbeing. Engagement in leisure activities that involved Socialising and Physical Activity positively predicted wellbeing. The profiles of predictors differed by measure: life satisfaction and negative affect were predicted only by personality traits, but positive affect, mental health and flourishing were affected by both personality traits and leisure activities. The cohorts only differed on one measure of wellbeing. Positive affect was lower in the middle age group and this coincided with lower levels of socialising activity in this age group. The study also examined the relationship between creativity and wellbeing. Negative affect was associated with greater engagement in creative hobbies and with greater creative potential. The study indicates the importance of jointly considering the influence of engaging in leisure activities in addition to personality traits when considering the wellbeing of adolescents and young adults. It highlights the importance of social and physical activities for positive affect, mental health and flourishing in this age group. It also sheds new light on the relationship between creativity and wellbeing in this young population.

## Supplementary Information

**Additional file 1.** Leisure Questionnaire

**Additional file 2.** Copy of Table 4 with exact *p* values

**Additional file 3.** Exploratory analysis including gender and cohort as predictors

## Data Availability

The datasets used and/or analysed during the current study are available from the corresponding author on reasonable request.

## Abbreviations

AUT: Alternate Uses Task
BFI-10: Big Five Inventory
MHC-SF: Mental Health Continuum-Short Form
OKC: Overcoming Knowledge Constraints

## Footnotes

1 Following the proposals made by Wetzels et al. [56] based on Jeffreys [57] the Bayesian findings were interpreted as follows.

I. Clear evidence for the alternate hypothesis (extremely strong evidence: BF_10_ > 100; very strong evidence: 30–100; strong evidence: 10–30; moderate evidence: 3–10);
II. Anecdotal evidence for the alternate hypothesis: BF_10_ = 1–3;
III. No evidence: BF_10_ = 1;
IV. Anecdotal evidence for the null hypothesis: BF_10_ = 0.3–1;
V. Clear evidence for the null hypothesis (moderate evidence: BF_10_ = 0.10–0.3; strong evidence: BF_10_ = 0.03–0.01; very strong evidence: BF_10_ = 0.01–0.03; extremely strong evidence: < 0.01)

.
