# Correction to: The role of personality traits and leisure activities in predicting wellbeing in young people

**DOI:** 10.1186/s40359-022-01011-3

**Published:** 2022-12-23

**Authors:** Sarah L. Asquith, Xu Wang, Daniel S. Quintana, Anna Abraham

**Affiliations:** 1 School of Humanities and Social Sciences, Leeds Beckett University, City Campus, Leeds, LS1 3HE UK; 2 Norwegian Centre for Mental Disorders Research (NORMENT), Division of Mental Health and Addiction, Oslo University Hospital & Institute of Clinical Medicine, University of Oslo, Oslo, Norway; 3 Department of Psychology, University of Oslo, Oslo, Norway; 4 NevSom, Department of Rare Disorders & Disabilities, Oslo University Hospital, Oslo, Norway; 5 Department of Educational Psychology, Frances Early College of Education, University of Georgia, Athens, USA; 6 Torrance Center of Creativity and Talent Development, Frances Early College of Education, University of Georgia, Athens, USA

**Correction to: BMC Psychology (2022) 10:249** 10.1186/s40359-022-00954-x

Following publication of the original article [1], some formatting and data entry errors were identified in Table 10, ‘Correlations between creativity and wellbeing variables’: The *p*-value for the correlation between overall originality and negative affect should not have been formatted in bold; The *p*-value for the correlation between OKC raw score and life satisfaction should not have been in bold either; The correlation between OKC raw score and mental health should be − 0.024; The correlation between negative affect and mental health should be − 0.490.

The table has been corrected in the original article, and a version of the table with the corrections highlighted can be seen in this erratum (Table 10).

**Table 10:**
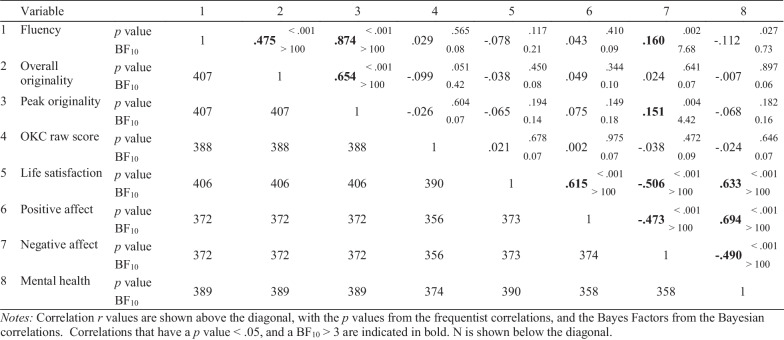
Correlations between creativity and wellbeing variables

The authors thank you for reading and apologise for any inconvenience caused.

## References

[1] Asquith SL, Wang X, Quintana DS, Abraham A (2022). The role of personality traits and leisure activities in predicting wellbeing in young people. BMC Psychol.

