# Supplementary material for: The role of personality traits and leisure activities in predicting wellbeing in young people
Source: BMC Psychol. 2022 Nov 4;10:249. doi: 10.1186/s40359-022-00954-x (PMC9636694; doi:10.1186/s40359-022-00954-x)
Supplement: Supplementary file 1 — Additional file 1. Leisure Questionnaire [file 40359_2022_954_MOESM1_ESM.docx]

Additional File 1

The Role of Personality Traits and Leisure Activities in Predicting Wellbeing in Young People – Leisure Questionnaire

## Leisure Questionnaire

We'd like you to tell us what you do when you're not at school, or doing school work. The questions will cover 5 areas:

Hobbies and interests Sports and physical activity Socialising Relaxing Work

Q1. Hobbies and interests. What hobbies or interests do you have?

|  | Thinking back over the last month, please tell us which ones you do and how often by ticking in the blue squares. | | | | | |  |  | For the activities that you do, please tick in the green squares to tell us who you do them with. Please tick all the boxes that apply. | | |
| --- | --- | --- | --- | --- | --- | --- | --- | --- | --- | --- | --- |
|  | Never | Less than once a week | 1-2 days a week | 3-4 days a week | 5-6 days a week | Every day |  |  | With a club, tutor or coach | With my friends or family | On my own |
| Playing a musical instrument | 🞏 | 🞏 | 🞏 | 🞏 | 🞏 | 🞏 |  |  | 🞏 | 🞏 | 🞏 |
| Art, drawing, painting | 🞏 | 🞏 | 🞏 | 🞏 | 🞏 | 🞏 |  |  | 🞏 | 🞏 | 🞏 |
| Drama | 🞏 | 🞏 | 🞏 | 🞏 | 🞏 | 🞏 |  |  | 🞏 | 🞏 | 🞏 |
| Singing | 🞏 | 🞏 | 🞏 | 🞏 | 🞏 | 🞏 |  |  | 🞏 | 🞏 | 🞏 |
| Crafts, making things | 🞏 | 🞏 | 🞏 | 🞏 | 🞏 | 🞏 |  |  | 🞏 | 🞏 | 🞏 |
| Photography or film-making | 🞏 | 🞏 | 🞏 | 🞏 | 🞏 | 🞏 |  |  | 🞏 | 🞏 | 🞏 |
| Writing music | 🞏 | 🞏 | 🞏 | 🞏 | 🞏 | 🞏 |  |  | 🞏 | 🞏 | 🞏 |
| Writing stories or poetry | 🞏 | 🞏 | 🞏 | 🞏 | 🞏 | 🞏 |  |  | 🞏 | 🞏 | 🞏 |
| Using a computer to create artworks or animation | 🞏 | 🞏 | 🞏 | 🞏 | 🞏 | 🞏 |  |  | 🞏 | 🞏 | 🞏 |
| Computer coding, or developing computer games | 🞏 | 🞏 | 🞏 | 🞏 | 🞏 | 🞏 |  |  | 🞏 | 🞏 | 🞏 |
| Making videos for YouTube channels | 🞏 | 🞏 | 🞏 | 🞏 | 🞏 | 🞏 |  |  | 🞏 | 🞏 | 🞏 |
| Other (please write in here) _______________________________ | 🞏 | 🞏 | 🞏 | 🞏 | 🞏 | 🞏 |  |  | 🞏 | 🞏 | 🞏 |
| Other please write in here) _______________________________ | 🞏 | 🞏 | 🞏 | 🞏 | 🞏 | 🞏 |  |  | 🞏 | 🞏 | 🞏 |
| Other (please write in here) _______________________________ | 🞏 | 🞏 | 🞏 | 🞏 | 🞏 | 🞏 |  |  | 🞏 | 🞏 | 🞏 |

Q2. Sports and physical activity.

| Please write in the sports and physical activities that you do | Thinking back over the last month, please tell us which ones you do and how often by ticking in the blue squares. | | | | | |  |  | For the activities that you do, please tick in the green squares to tell us who you do them with. Please tick all the boxes that apply. | | |
| --- | --- | --- | --- | --- | --- | --- | --- | --- | --- | --- | --- |
|  | Never | Less than once a week | 1-2 days a week | 3-4 days a week | 5-6 days a week | Every day |  |  | With a club, tutor or coach | With my friends or family | On my own |
|  | 🞏 | 🞏 | 🞏 | 🞏 | 🞏 | 🞏 |  |  | 🞏 | 🞏 | 🞏 |
|  | 🞏 | 🞏 | 🞏 | 🞏 | 🞏 | 🞏 |  |  | 🞏 | 🞏 | 🞏 |
|  | 🞏 | 🞏 | 🞏 | 🞏 | 🞏 | 🞏 |  |  | 🞏 | 🞏 | 🞏 |
|  | 🞏 | 🞏 | 🞏 | 🞏 | 🞏 | 🞏 |  |  | 🞏 | 🞏 | 🞏 |
|  | 🞏 | 🞏 | 🞏 | 🞏 | 🞏 | 🞏 |  |  | 🞏 | 🞏 | 🞏 |
|  | 🞏 | 🞏 | 🞏 | 🞏 | 🞏 | 🞏 |  |  | 🞏 | 🞏 | 🞏 |
|  | 🞏 | 🞏 | 🞏 | 🞏 | 🞏 | 🞏 |  |  | 🞏 | 🞏 | 🞏 |
|  | 🞏 | 🞏 | 🞏 | 🞏 | 🞏 | 🞏 |  |  | 🞏 | 🞏 | 🞏 |

Q3. Socialising. Thinking back over the last month, please tell us what you do when you're spending time with your friends or family, and tell us how often you do it, by ticking the squares in the blue box.

|  |  |  | | | | |
| --- | --- | --- | --- | --- | --- | --- |
|  | Never | Less than once a week | 1-2 days a week | 3-4 days a week | 5-6 days a week | Every day |
| Hanging out with your friends at your/their homes | 🞏 | 🞏 | 🞏 | 🞏 | 🞏 | 🞏 |
| Shopping | 🞏 | 🞏 | 🞏 | 🞏 | 🞏 | 🞏 |
| Going out for drinks or food | 🞏 | 🞏 | 🞏 | 🞏 | 🞏 | 🞏 |
| Going out to listen to music or see a band | 🞏 | 🞏 | 🞏 | 🞏 | 🞏 | 🞏 |
| Going to sports events | 🞏 | 🞏 | 🞏 | 🞏 | 🞏 | 🞏 |
| Going to the cinema or the theatre | 🞏 | 🞏 | 🞏 | 🞏 | 🞏 | 🞏 |
| Visiting places with your family | 🞏 | 🞏 | 🞏 | 🞏 | 🞏 | 🞏 |
| Visiting family or friends | 🞏 | 🞏 | 🞏 | 🞏 | 🞏 | 🞏 |
| Attending church, mosque, temple or synagogue | 🞏 | 🞏 | 🞏 | 🞏 | 🞏 | 🞏 |
| Other (please write in here _________________) | 🞏 | 🞏 | 🞏 | 🞏 | 🞏 | 🞏 |
| Other (please write in here _________________) | 🞏 | 🞏 | 🞏 | 🞏 | 🞏 | 🞏 |
| Other (please write in here _________________) | 🞏 | 🞏 | 🞏 | 🞏 | 🞏 | 🞏 |

Q4. Relaxing. Thinking back over the last month, please tell what you like to do when you're relaxing at home, and tell us how often you do it, by ticking the squares in the blue box.

|  | Never | Less than once a week | 1-2 days a week | 3-4 days a week | 5-6 days a week | Every day |
| --- | --- | --- | --- | --- | --- | --- |
| Listening to music | 🞏 | 🞏 | 🞏 | 🞏 | 🞏 | 🞏 |
| Watching programmes on TV, Netflix, YouTube etc. | 🞏 | 🞏 | 🞏 | 🞏 | 🞏 | 🞏 |
| Playing computer games | 🞏 | 🞏 | 🞏 | 🞏 | 🞏 | 🞏 |
| Playing board games | 🞏 | 🞏 | 🞏 | 🞏 | 🞏 | 🞏 |
| Reading | 🞏 | 🞏 | 🞏 | 🞏 | 🞏 | 🞏 |
| On my smartphone/the internet | 🞏 | 🞏 | 🞏 | 🞏 | 🞏 | 🞏 |
| Other (please write in here _________________) | 🞏 | 🞏 | 🞏 | 🞏 | 🞏 | 🞏 |
| Other (please write in here _________________) | 🞏 | 🞏 | 🞏 | 🞏 | 🞏 | 🞏 |

| Q5. Do you have a job? |
| --- |
| Please tell us what you do:__________________________________________________________________________________________  Please tell us how many hours a week you work: ______________________________________________________________________  If you have more than one job, you can add them in below: |

Please tell us what you do: _____________________________________________________________________________________________

Please tell us how many hours a week you work: ___________________________________________________________________________

Please tell us what you do: _____________________________________________________________________________________________

Please tell us how many hours a week you work: ___________________________________________________________________________

| Q6. Is there anything else that you like to do that you haven't had the opportunity to tell us about? If so, please tell us what it is, & how often you do it. |
| --- |
