# Supplementary material for: The role of personality traits and leisure activities in predicting wellbeing in young people
Source: BMC Psychol. 2022 Nov 4;10:249. doi: 10.1186/s40359-022-00954-x (PMC9636694; doi:10.1186/s40359-022-00954-x)
Supplement: Supplementary file 2 — Additional file 2. Copy of Table 4 with exact p values [file 40359_2022_954_MOESM2_ESM.docx]

Additional File 2

The Role of Personality Traits and Leisure Activities in Predicting Wellbeing in Young People – copy of Table 4

| **Table 4** *Correlations between wellbeing, personality trait and leisure activities variables* | | | | | | | | | | | | | | | | | | | | | | | | | | | | |
| --- | --- | --- | --- | --- | --- | --- | --- | --- | --- | --- | --- | --- | --- | --- | --- | --- | --- | --- | --- | --- | --- | --- | --- | --- | --- | --- | --- | --- |
|  |  |  | 1 | 2 | | 3 | | 4 | | 5 | | 6 | | 7 | | 8 | | 9 | | 10 | | 11 | | 12 | | 13 | | |
| 1 | Life satis-faction | *p* | 1 | **.615** | < .001 | **-.506** | < .001 | **.633** | < .001 | **.168** | < .001 | **.247** | < .001 | **.205** | < .001 | **-.300** | < .001 | -.133 | .007 | -.075 | .209 | .096 | .112 | .156 | .009 | -.087 | .150 | |
|  |  | BF_10_ |  |  | > 100 |  | > 100 |  | > 100 |  | > 100 |  | > 100 |  | > 100 |  | > 100 |  | 2.31 |  | 0.16 |  | 0.26 |  | 2.20 |  | 0.21 | |
| 2 | Positive affect | *p* | 373 | 1 |  | **-.473** | < .001 | **.694** | < .001 | **.265** | < .001 | **.192** | < .001 | **.151** | .004 | **-.326** | < .001 | -.013 | .808 | .000 | .996 | .162 | .009 | **.247** | < .001 | .003 | .964 | |
|  |  | BF_10_ |  |  |  |  | > 100 |  | > 100 |  | > 100 |  | 65.13 |  | 4.35 |  | > 100 |  | 0.07 |  | 0.08 |  | 2.30 |  | > 100 |  | 0.08 | |
| 3 | Negative affect | *p* | 373 | 374 |  | 1 |  | **-.490** | < .001 | **-.181** | < .001 | **-.259** | < .001 | **-.220** | < .001 | **-.366** | < .001 | **.203** | < .001 | .125 | .044 | -.149 | .017 | -.158 | .011 | .068 | .275 | |
|  |  | BF_10_ |  |  |  |  |  |  | > 100 |  | 30.35 |  | > 100 |  | > 100 |  | > 100 |  | > 100 |  | 0.58 |  | 1.23 |  | 1.93 |  | 0.14 | |
| 4 | Mental health | *p* | 390 | 358 |  | 358 |  | 1 |  | **.350** | < .001 | **.260** | < .001 | **.225** | < .001 | **-.381** | < .001 | -.047 | .354 | .012 | .838 | .160 | .009 | **.304** | < .001 | .001 | .990 | |
|  |  | BF_10_ |  |  |  |  |  |  |  |  | > 100 |  | > 100 |  | > 100 |  | > 100 |  | 0.10 |  | 0.08 |  | 2.35 |  | > 100 |  | 0.08 | |
| 5 | Extraversion | *p* | 407 | 373 |  | 373 |  | 390 |  | 1 |  | .099 | .046 | .037 | .453 | **-.381** | < .001 | .006 | .898 | -.044 | .462 | .131 | .030 | **.296** | < .001 | .004 | .948 | |
|  |  | BF_10_ |  |  |  |  |  |  |  |  |  |  | 0.45 |  | 0.08 |  | > 100 |  | 0.06 |  | 0.10 |  | 0.79 |  | > 100 |  | 0.08 | |
| 6 | Agreeableness | *p* | 404 | 370 |  | 370 |  | 389 |  | 405 |  | 1 |  | .**179** | < .001 | -.115 | .021 | -.041 | .406 | .067 | .264 | .066 | .275 | .060 | .325 | -.064 | .287 | |
|  |  | BF_10_ |  |  |  |  |  |  |  |  |  |  |  |  | 41.63 |  | 0.88 |  | 0.09 |  | 0.14 |  | 0.14 |  | 0.12 |  | 0.13 | |
| 7 | Conscient-iousness | *p* | 404 | 370 |  | 370 |  | 388 |  | 404 |  | 402 |  | 1 |  | -.002 | .969 | -.079 | .111 | -.061 | .311 | .132 | .029 | .040 | .508 | -.116 | .054 | |
|  |  | BF_10_ |  |  |  |  |  |  |  |  |  |  |  |  |  |  | 0.62 |  | 0.02 |  | 0.13 |  | 0.82 |  | 0.09 |  | 0.48 | |
| 8 | Neuroticism | *p* | 405 | 371 |  | 371 |  | 388 |  | 405 |  | 402 |  | 405 |  | 1 |  | .006 | .904 | .025 | .679 | -.174 | .004 | **-.167** | .005 | .028 | .646 | |
|  |  | BF_10_ |  |  |  |  |  |  |  |  |  |  |  |  |  |  |  |  | 0.06 |  | 0.08 |  | 4.79 |  | 3.50 |  | 0.08 | |
| 9 | Openness | *p* | 408 | 374 |  | 374 |  | 391 |  | 408 |  | 406 |  | 405 |  | 406 |  | 1 |  | **.394** | < .001 | .083 | .166 | -.093 | .123 | **.192** | .001 | |
|  |  | BF_10_ |  |  |  |  |  |  |  |  |  |  |  |  |  |  |  |  |  |  | > 100 |  | 0.20 |  | 0.25 |  | 12.93 | |
| 10 | Creative hobbies | *p* | 280 | 260 |  | 260 |  | 273 |  | 280 |  | 278 |  | 278 |  | 278 |  | 281 |  | 1 |  | **.183** | .002 | .053 | .382 | **.306** | < .001 | |
|  |  | BF_10_ |  |  |  |  |  |  |  |  |  |  |  |  |  |  |  |  |  |  |  |  | 7.86 |  | 0.11 |  | > 100 | |
| 11 | Physical activity | *p* | 277 | 257 |  | 257 |  | 270 |  | 277 |  | 275 |  | 275 |  | 275 |  | 278 |  | 277 |  | 1 |  | .156 | .009 | .136 | .024 | |
|  |  | BF_10_ |  |  |  |  |  |  |  |  |  |  |  |  |  |  |  |  |  |  |  |  |  |  | 2.20 |  | 0.95 | |
| 12 | Socialising | *p* | 277 | 257 |  | 257 |  | 270 |  | 277 |  | 275 |  | 275 |  | 275 |  | 278 |  | 276 |  | 277 |  | 1 |  | .149 | .013 | |
|  |  | BF_10_ |  |  |  |  |  |  |  |  |  |  |  |  |  |  |  |  |  |  |  |  |  |  |  |  | 1.62 | |
| 13 | Sedentary activities |  | 277 | 257 |  | 257 |  | 270 |  | 277 |  | 275 |  | 275 |  | 275 |  | 278 |  | 276 |  | 277 |  | 278 |  | 1 |  | |
| *Notes*: Correlation r values are shown above the diagonal, with the p values from the frequentist correlations, and the Bayes Factors from the Bayesian correlations. Correlations that have a p value < .05, and a BF10 > 3 are indicated in bold. N is shown below the diagonal. | | | | | | | | | | | | | | | | | | | | | | | | | | | |  |
