# Supplementary material for: The role of personality traits and leisure activities in predicting wellbeing in young people
Source: BMC Psychol. 2022 Nov 4;10:249. doi: 10.1186/s40359-022-00954-x (PMC9636694; doi:10.1186/s40359-022-00954-x)
Supplement: Supplementary file 3 — Additional file 3. Exploratory analysis including gender and cohort as predictors [file 40359_2022_954_MOESM3_ESM.docx]

Additional File 3

Exploratory analyses of predictors of wellbeing including gender and cohort (as a proxy for age) as predictors.

**Approach to Statistical Analysis**

For full details of the method, please refer to the main paper. Here we report post-hoc exploratory analyses which include gender and age as predictors. Gender is a categorical variable with three levels, male, female, and non-binary. As there was only one participant who identified as non-binary, this category was excluded from this exploratory analysis. We collected information on age from participants in the study, but as they were recruited in three cohorts, age was not a continuous variable. We have therefore used cohort as a proxy for age, using dummy coding. In the next section we report the results of the frequentist regression analysis. As Bayesian linear regression in JASP does not currently allow the inclusion of categorical predictors, we were not able to carry out this analysis.

**Results**

The findings (see Tables S6a and S6b) indicated that the significant predictors of life satisfaction, negative affect and mental health were unchanged when gender and cohort were included. For positive affect there were some differences in the significant predictors. Gender was a significant predictor (*p* = .008), such that identifying as female predicted higher levels of positive affect. Openness became a significant predictor of positive affect (*p* = .024), such that higher levels of openness were associated with lower positive affect and socialising was no longer a significant predictor (*p* = .054). The predictors of flourishing were unchanged, and extraversion became a significant predictor of languishing (*p* = .042), such that higher levels of extraversion predicted lower odds of languishing.

| **Table S6a** *Summary of the frequentist multiple regressions predicting wellbeing from personality and leisure activities, including gender and cohort as predictors* | | | | | | | | | | | | | | | | | | | | | | | | | | | | | | | | | | | |  |
| --- | --- | --- | --- | --- | --- | --- | --- | --- | --- | --- | --- | --- | --- | --- | --- | --- | --- | --- | --- | --- | --- | --- | --- | --- | --- | --- | --- | --- | --- | --- | --- | --- | --- | --- | --- | --- |
|  | Life satisfaction | | | | | |  | | | Positive affect | | | | |  | Negative affect | | | | | | | | |  | | Mental health | | | | | | | | |  |
| Predictor | B | 95% CI | | β | |  | | | B | | | 95% CI | | β | | | |  | | B | | 95% CI | | β | | | | | B | | 95% CI | | β | | | |
| Gender | 0.24† | [-0.03, 0.50] | .10 | |  | | | 1.60** | | | [0.41, 2.78] | | .16 | | | |  | | -1.09 | | [-2.41, 0.24] | | -.10 | | |  | | 0.06 | | [-0.20, 0.32] | | .03 | | |  |  |
| Cohort 1 vs Cohort 2 | 0.12 | [-0.03, 0.37] | .07 | |  | | | -0.86 | | | [-2.99, 0.28] | | -.11 | | | |  | | 0.18 | | [-1.08, 1.45] | | .02 | | |  | | -0.02 | | [-0.26, 0.23] | | -.01 | | |  |  |
| Cohort 1 vs Cohort 3 | 0.24 | [-0.13, 0.53] | .12 | |  | | | 0.44 | | | [-0.88, 1.76] | | .05 | | | |  | | -0.38 | | [-1.86, 1.10] | | -.04 | | |  | | 0.17 | | [-0.12, 0.45] | | .08 | | |  |  |
| Extraversion | 0.01 | [-0.06, 0.06] | .03 | |  | | | 0.11 | | | [-0.11, 0.33] | | .06 | | | |  | | 0.02 | | [-0.22, 0.27] | | .01 | | |  | | 0.06* | | [0.01, 0.11] | | .15 | | |  |  |
| Agreeableness | 0.09** | [0.04, 0.15] | .17 | |  | | | 0.17 | | | [-0.10, 0.44] | | .07 | | | |  | | -0.54*** | | [-0.84, -0.23] | | -.19 | | |  | | 0.07* | | [0.01, 0.13] | | .13 | | |  |  |
| Conscientiousness | 0.08** | [0.02, 0.13] | .15 | |  | | | 0.32* | | | [0.07, 0.57] | | .14 | | | |  | | -0.40** | | [-0.69, -0.12] | | -.16 | | |  | | 0.11*** | | [0.06, 0.17] | | .22 | | |  |  |
| Neuroticism | -0.14*** | [-0.20, -0.09] | -.33 | |  | | | -0.66*** | | | [-0.90, -0.43] | | -.36 | | | |  | | 0.85*** | | [0.58, 1.11] | | .39 | | |  | | -0.13*** | | [-0.19, -0.08] | | -.31 | | |  |  |
| Openness | -0.09*** | [-0.14, -0.04] | -.21 | |  | | | -0.24* | | | [-0.44, -0.03] | | -.13 | | | |  | | 0.53*** | | [0.30, 0.76] | | .25 | | |  | | -0.03 | | [-0.08, 0.01] | | -.08 | | |  |  |
| Physical activity | 0.01 | [-0.01, 0.03] | .06 | |  | | | 0.06 | | | [-0.03, 0.15] | | .08 | | | |  | | -0.08 | | [-0.18, 0.02] | | -.09 | | |  | | 0.01 | | [-0.01, 0.03] | | .04 | | |  |  |
| Socialising | 0.01 | [-0.01, 0.03] | .08 | |  | | | 0.08† | | | [0.00, 0.15] | | .12 | | | |  | | -0.03 | | [-0.12, 0.06] | | -.04 | | |  | | 0.03** | | [0.01, 0.04] | | .18 | | |  |  |
| Sedentary activities | -0.00 | [-0.02, 0.01] | -.02 | |  | | | 0.03 | | | [-0.05, 0.10] | | .04 | | | |  | | -0.02 | | [-0.10, 0.06] | | -.03 | | |  | | 0.00 | | [-0.01, 0.02] | | .03 | | |  |  |
| *R^2^* | .26 |  |  | |  | | | .28 | | |  | |  | | | |  | | .32 | |  | |  | | |  | | .33 | |  | |  | | |  |  |
| *F* | 8.02 |  |  | |  | | | 8.28 | | |  | |  | | | |  | | 10.17 | |  | |  | | |  | | 11.33 | |  | |  | | |  |  |
| *p* | < .001 |  |  | |  | | | < .001 | | |  | |  | | | |  | | < .001 | |  | |  | | |  | | < .001 | |  | |  | | |  |  |
| N | 268 |  |  | |  | | | 248 | | |  | |  | | | |  | | 248 | |  | |  | | |  | | 262 | |  | |  | | |  |  |
| *Note*: Gender is coded as a binary variable (0 = male, 1 = female). † *p* < .10. **p* < .05. ***p* < .01. ****p* < .001. | | | | | | | | | | | | | | | | | | | | | | | | | | | | | | | | | |  |  |  |

| **Table S6b** *Summary of the frequentist nominal regressions predicting flourishing and languishing from personality and leisure activities, including gender and cohort as predictors* | | | | | | | | | | |
| --- | --- | --- | --- | --- | --- | --- | --- | --- | --- | --- |
|  | Flourishing | | | |  | Languishing | | | | |
|  | 95% CI for Odds Ratio | | | |  | 95% CI for Odds Ratio | | | | |
| Predictor | b (SE) | Odds Ratio | Lower | Upper |  | b (SE) | Odds Ratio | Lower | Upper |  |
| Gender | 0.88 (0.47) | 2.41 | 0.97 | 6.00 |  | 0.07 (0.65) | 1.07 | 0.30 | 3.82 |  |
| Cohort 1 vs Cohort 2 | 0.27 (0.47) | 1.31 | 0.52 | 3.27 |  | 0.53 (0.56) | 1.70 | 0.57 | 5.04 |  |
| Cohort 1 vs Cohort 3 | -1.01 (0.53) | 0.36 | 0.13 | 1.03 |  | 0.25 (0.65) | 1.28 | 0.36 | 4.57 |  |
| Extraversion | 0.12 (0.10) | 1.12 | 0.92 | 1.36 |  | -0.21 (0.10)* | 0.81 | 0.66 | 0.99 |  |
| Agreeableness | 0.17 (0.13) | 1.18 | 0.93 | 1.52 |  | -0.08 (0.13) | 0.93 | 0.72 | 1.19 |  |
| Conscientiousness | 0.38 (0.11)*** | 1.46 | 1.17 | 1.82 |  | -0.26 (0.14)† | 0.77 | 0.59 | 1.01 |  |
| Neuroticism | -0.15 (0.10) | 0.86 | 0.70 | 1.05 |  | 0.20 (0.12) | 1.23 | 0.96 | 1.56 |  |
| Openness | -0.02 (0.09) | 0.98 | 0.82 | 1.16 |  | 0.03 (0.10) | 1.03 | 0.85 | 1.25 |  |
| Physical activity | 0.09 (0.04)* | 1.09 | 1.01 | 1.17 |  | 0.04 (0.05) | 1.04 | 0.95 | 1.14 |  |
| Socialising | 0.02 (0.03) | 1.02 | 0.96 | 1.09 |  | -0.06 (0.05) | 0.94 | 0.86 | 1.03 |  |
| Sedentary activities | 0.09 (0.03)* | 1.09 | 1.02 | 1.17 |  | 0.06 (0.04)† | 1.06 | 0.99 | 1.14 |  |
| *Note*: The multinomial logistic regression was run with “moderately mentally healthy” as the reference category. Gender is coded as a binary variable (0 = male, 1 = female).  *R^2^* = .28 (Cox & Snell), .34 (Nagelkerke). Model χ^2^(22) = 86.90, *p* < .001, N = 263.  † *p* < .10. **p* < .05. ***p* < .01. ****p* < .001. | | | | | | | | | | |
